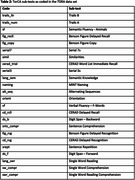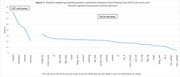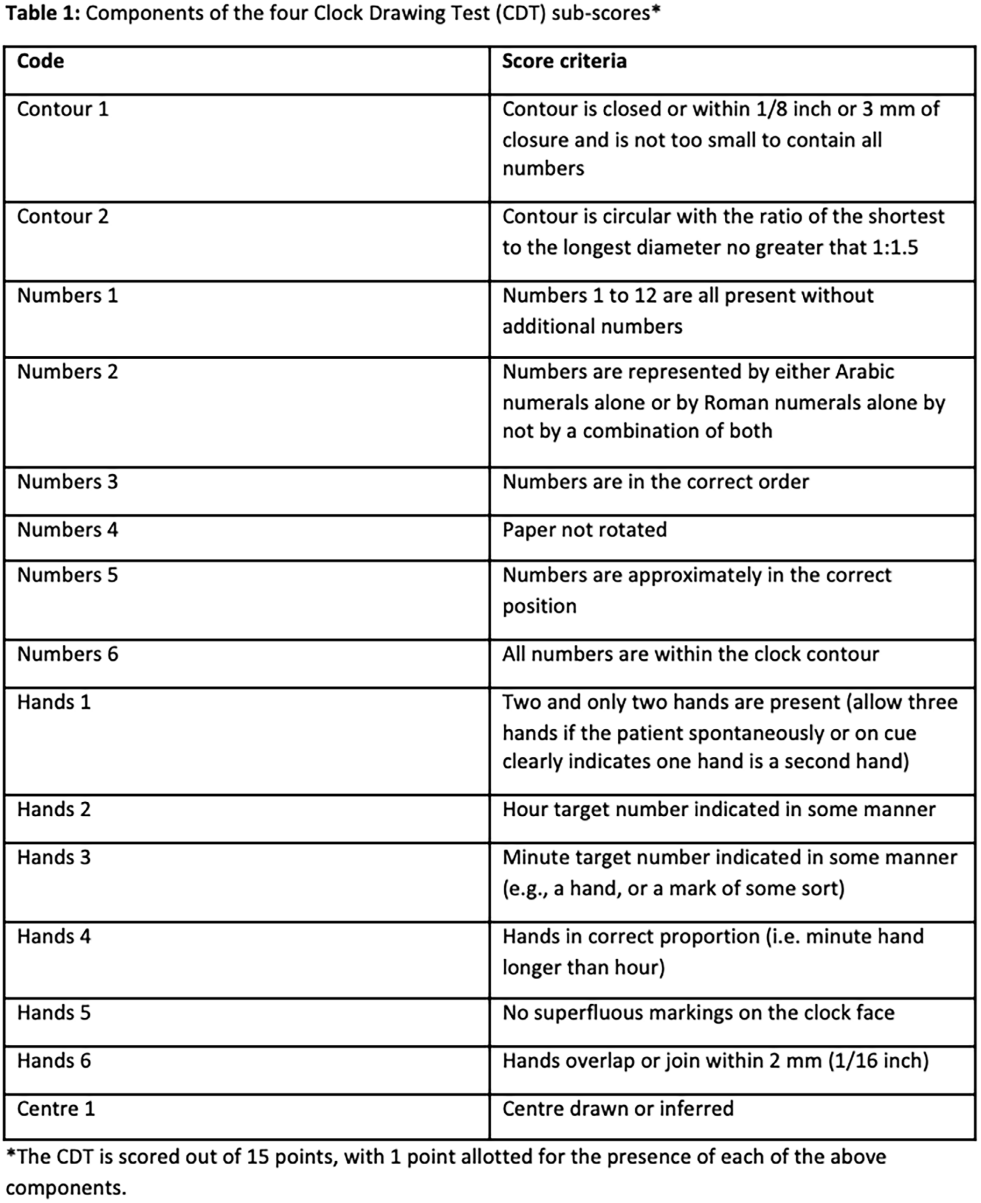# Clock Drawing as a Tool to Reduce Cognitive Assessment Time

**DOI:** 10.1002/alz70861_108482

**Published:** 2025-12-23

**Authors:** Carolyn Pavoni, Nodee Chowdhury, Malcolm Binns, Sandra E. Black, Sanjeev Kumar, David F. Tang‐Wai, Carmela Tartaglia, Morris Freedman

**Affiliations:** ^1^ Baycrest Health Sciences, Toronto, ON Canada; ^2^ University of Toronto, Toronto, ON Canada; ^3^ Dalla Lana School of Public Health, University of Toronto, Toronto, ON Canada; ^4^ Rotman Research Institute, Baycrest Academy for Research and Education, Toronto, ON Canada; ^5^ Baycrest Academy for Research and Education, Toronto, ON Canada; ^6^ Sunnybrook Research Institute, Toronto, ON Canada; ^7^ Toronto Dementia Research Alliance, Toronto, ON Canada; ^8^ Centre for Addiction and Mental Health, Toronto, ON Canada; ^9^ Department of Medicine, University of Toronto, Toronto, ON Canada; ^10^ Memory Clinic, Toronto Western Hospital, University Health Network, Toronto, ON Canada; ^11^ Tanz Centre for Research in Neurodegenerative Diseases, University of Toronto, Toronto, ON Canada; ^12^ Toronto Dementia Research Alliance (TDRA), Toronto, ON Canada; ^13^ Mt. Sinai Hospital, Toronto, ON Canada; ^14^ Rotman Research Institute at Baycrest, Toronto, ON Canada; ^15^ Rotman Research Institute, Baycrest Health Sciences, Toronto, ON Canada

## Abstract

**Background:**

The Clock Drawing Test (CDT) is a widely used neuropsychological tool and a component of the Toronto Cognitive Assessment (TorCA). We aimed to determine whether specific CDT sub‐scores predict performance on other TorCA sub‐tests. Identifying such relationships may support removal of measures, thereby reducing overall TorCA administration time.

**Method:**

Data were obtained from the Toronto Dementia Research Alliance database which includes patient demographic and clinical information assessed at four Toronto area memory clinics. Performance on the CDT is based upon 4 sub‐scores: contour, numbers, hands, and centre. The TorCA contains 24 sub‐tests in addition to the CDT that evaluate cognitive domains including memory, visuospatial, working memory/attention/executive control, and language. To identify a linear combination of CDT sub‐scores that is maximally associated with a linear combination of the other TorCA sub‐tests, we used singular value decomposition of their cross‐block correlation matrix as applied by Partial Least Squares. Reported saliences describe relative contributions of each variable to the linear combinations. Saliences greater than 2 standard errors (se) are considered significant.

**Result:**

CDT and TorCA sub‐test saliences using data from 1,872 participants are presented in Figure 1. The “hand” sub‐score returned relatively large salience (0.71, se = 0.31). The “numbers” sub‐score had slightly lower salience (0.50, se = 0.22); the “centre” sub‐score returned a similar salience (0.43, se = 0.19). TorCA sub‐tests of working memory/attention/executive control, semantic knowledge, and visuospatial function returned larger saliences (Figure 1). Specifically, the strongest association was with Trails B (0.33, se = 0.15), followed by Trails A (0.27, se = 0.13), semantic fluency (0.25, se = 0.11), Benson Figure copy (0.25, se = 0.12) and recall (0.25, se = 0.11).

**Conclusion:**

Within the CDT scoring system, clock hands and numbers were the strongest predictors of performance on the TorCA sub‐tests. The most robust associations were with domains of working memory/attention/executive function, semantic knowledge, and visuospatial function. We plan to apply artificial intelligence to classify clocks based on these cognitive functions. We will then examine how these classified clocks relate to TorCA sub‐tests to determine whether redundant tests could be removed, thereby shortening administration time.